# Gene-Expressing Liposomes as Synthetic Cells for Molecular Communication Studies

**DOI:** 10.3389/fbioe.2019.00001

**Published:** 2019-01-17

**Authors:** Giordano Rampioni, Francesca D'Angelo, Livia Leoni, Pasquale Stano

**Affiliations:** ^1^Department of Science, University Roma Tre, Rome, Italy; ^2^Department of Biological and Environmental Sciences and Technologies (DiSTeBA), University of Salento, Lecce, Italy

**Keywords:** synthetic cells, bottom-up synthetic biology, molecular communications, quorum sensing, lipid vesicles (liposomes), cell-free protein synthesis

## Abstract

The bottom-up branch of synthetic biology includes—among others—innovative studies that combine cell-free protein synthesis with liposome technology to generate cell-like systems of minimal complexity, often referred to as synthetic cells. The functions of this type of synthetic cell derive from gene expression, hence they can be programmed in a modular, progressive and customizable manner by means of *ad hoc* designed genetic circuits. This experimental scenario is rapidly expanding and synthetic cell research already counts numerous successes. Here, we present a review focused on the exchange of chemical signals between liposome-based synthetic cells (operating by gene expression) and biological cells, as well as between two populations of synthetic cells. The review includes a short presentation of the “molecular communication technologies,” briefly discussing their promises and challenges.

## Molecular Communications and Synthetic Cells (SCs)

Natural organisms coordinate their activities through communication. Isolated cells, tissue cells, as well as higher organisms, share their environment with other living forms. Tactile, physical, and especially chemical signals define in unique and complex manner the sensory world of living beings. Communications in the chemical domain are ubiquitous intercellular processes, and play important roles in all organisms.

**Figure 1:**
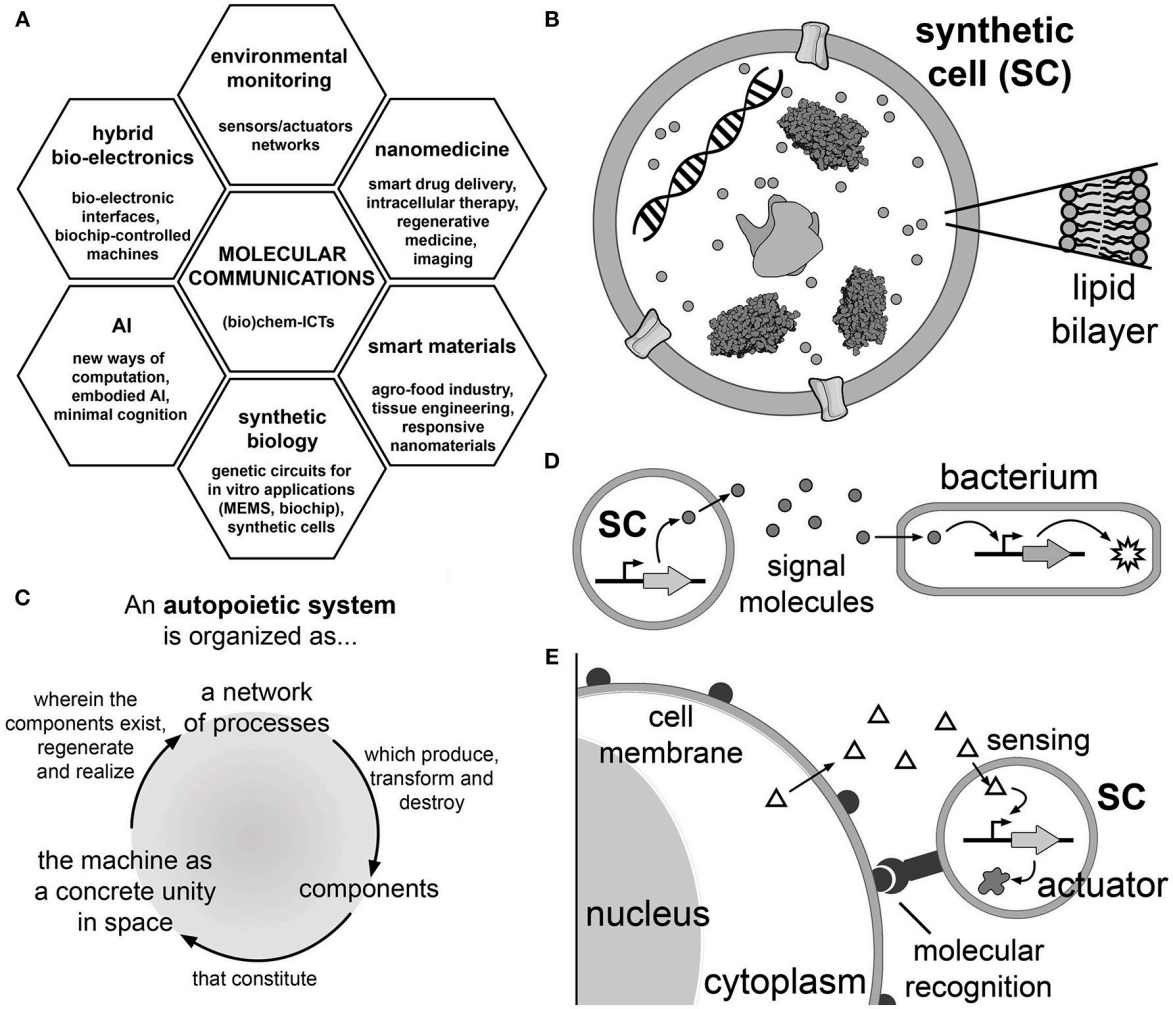
Molecular communications based on synthetic cell (SC) technology. **(A)** Application areas of molecular communication research. Molecular communication is a (bio)chem-information and communication technology that can be applied to nanomedicine (smart drug delivery systems), smart responsive materials, synthetic biology (construction of biochips), artificial intelligence (AI), hybrid bio-electronic systems and for sensors in environmental monitoring (Nakano et al., 2013). **(B)** Synthetic cells are cell-like systems, generally built by encapsulating a number of (bio)molecular components into artificial micro-compartments. One of the possible designs focuses on liposome-based SCs operating by gene expression (Luisi, 2002; Luisi et al., 2006). With this aim, TX-TL kits produce the protein(s) of interest starting from the corresponding DNA sequence. The SC membrane can be functionalized with membrane proteins as pores (Noireaux and Libchaber, 2004) and receptors (Hamada et al., 2014); cytoskeletal proteins have been implemented as well (Maeda et al., 2012). **(C)** The principles of autopoiesis (self-production) (Varela et al., 1974), which guides the long-term goal of constructing SCs that produce all their components. Autopoiesis provides insights into the spatial and dynamical organization that a chemical system should be endowed with in order to display self-maintenance, organizational closure, homeostasis and reproduction achieved by the internal processes of manufacturing and assembling its components. **(D)** Schematic representation of a SC which produces and releases a signal molecule into the environment. The signal is perceived by a natural cell (e.g., a bacterium) that consequently activates a response (for example, a reporter protein, an enzyme operating as an actuator to perform a certain operation, including a reply signaling) (Nakano et al., 2011; Stano et al., 2012). Table 1 reports several cases of unidirectional or bidirectional molecular communications between SCs, or between SCs and natural cells. **(E)** The vision of using SCs as smart drug delivery systems or for enzyme replacement therapy (Leduc et al., 2007). SCs, intended as a biotechnological evolution of current liposomes for drug delivery, reach and bind to the target cells by a molecular recognition mechanism and activate their internal circuits responding to chemical stimuli and consequently act, in a programmable manner, for a certain task (e.g., producing a therapeutic or diagnostic agent Ding et al., 2018; Krinsky et al., 2018, or a secondary easy-to-detect signal, etc.). The chemical stimulus can be an endogenous chemical that derives from the target cell itself (as shown in the cartoon) or from other tissues (not shown), as well as purposely-added exogenous chemicals (not shown).

Inspired by the already mentioned capabilities of natural organisms, a new branch of biomimetic technology has been proposed which focuses on *molecular communications* (Nakano et al., 2011, 2013). Network engineers have envisioned the exploitation of chemical exchanges as the basis for developing new types of Information and Communication Technologies (the so-called bio-chem-ICTs, Figure 1A). This is an exciting new arena for engineers and biologists that aims at the construction of well-characterized biological parts, devices, and systems that will process chemical information in a controlled and programmable manner, as it happens with classical electric signals. The challenge, here, relies on the ability of managing communication and information processing through chemical signals with the same mastery as nature has done for billions of years. Such a broad and innovative territory of research offers several opportunities for various approaches to synthetic biology, which needs adequate theoretical frameworks, numerical modeling strategies, and experimental methodologies. More generally, bio-chem-ICTs refers to radically new forms of computation, communication, and information processing approaches—at the nano- and micro-scale levels—based on chemical and biochemical systems (Amos et al., 2011).

Owing to our direct involvement in the field (Stano et al., 2012; Rampioni et al., 2014, 2018), and considering recent exciting reports, in this review we present and discuss the intersection between the bio-chem-ICT idea of exchanging chemical signals in a programmable way, and the bottom-up synthetic biology approach focused on the construction of cell-like systems based on gene expression inside liposomes (Luisi, 2002; Noireaux and Libchaber, 2004; Luisi et al., 2006; Ichihashi et al., 2010; Stano et al., 2011; Nourian and Danelon, 2013; Spencer et al., 2013). For simplicity, we will shortly refer to these systems simply as “synthetic cells” (SCs, Figure 1B), keeping in mind that these are rather simple mimics of biological cells.

**Table 1:** Comparative analysis of six experimental reports based on liposome SCs that send/receive chemical signals (2009–2018).

**#**	**1**	**2**	**3**	**4**		**5**	**6**	**7**
Reference No.	Gardner et al., 2009	Lentini et al., 2014	Adamala et al., 2017	Lentini et al., 2017		Tang et al., 2018	Rampioni et al., 2018	Ding et al., 2018
Year	2009	2014	2017	2017		2017	2018	2018
Directionality	SCs → bacteria	SCs → bacteria	SCs → SCs	bacteria → SCs	SCs → bacteria	SCs → SCs	SCs → bacteria	bacteria → SCs
Sender	SCs: DPhPC vesicles encapsulating the reagents of the formose reaction	SCs:POPC/chol vesicles encapsulating the PURE system	SCs: POPC/chol vesicles encapsulating *E. coli* extracts	Bacteria:*V. fischeri*	SCs: POPC/chol vesicles encapsulating *E. coli* extracts	SCs:POPC/chol vesicles encapsulating the PURE system	SCs: POPC vesicles encapsulating the PURE system	Bacteria:*E. coli*
Sender details (specifications, preparation…)	Thin film hydration, sonication, freezing-thawing, extrusion (100 nm)	Hydration of freeze-dried homogeneized liposomes	Hydration of freeze-dried extruded (1 μm) liposomes		Hydration of freeze-dried liposomes	Hydration of freeze-dried extruded (1 μm) liposomes	Droplet transfer method (2–5 μm)	Transformed with a plasmid encoding for EsaI under T7 promoter
Production of signal molecule?	Yes	No	No	Yes	Yes	No	Yes	Yes
Actuator(s) for the production of signals or for its emission	Formose reaction	α-Haemolysin (pore formation) produced by gene expression as a response to theophylline	α-Haemolysin (pore formation), produced by gene expression as a response to arabinose or theophylline	LuxI synthase produced by the cellular own gene	*E. coli* extract converts C2-CoA into longer fatty acid chains LuxI, produced by gene expression synthesizes the signal molecule	α-Haemolysin (pore formation), produced by gene expression as a response to 3OC6-HSL	RhlI, produced by gene expression, synthesizes the signal molecule	EsaI, produced by gene expression
Precursors	Formaldehyde, borate	None	None	Cellular metabolite precursors	SAM, C2-CoA	None	SAM, C4-CoA	Cellular metabolite precursors
Signal molecule	AI-2-like borate-esters of formose products	IPTG	IPTG (or doxycycline)	3OC6-HSL	HSLs molecules	Glucose	C4-HSL	HSL molecules
Exporting system	α-Haemolysin	α-Haemolysin	α-Haemolysin	None (diffusion through the bacterial membrane)	None (diffusion through the vesicle membrane)	α-Haemolysin	None (diffusion through the vesicle membrane)	None (diffusion through the bacterial membrane)
Medium	Liquid LB medium	Liquid M9 minimal medium	Liquid HEPES buffer including KCl and MgCl_2_	Liquid LBS medium	Liquid LBS	Liquid HEPES buffer	Gel LB-Agar	Liquid ACB buffer
Receiver	Bacteria: *V. harveyi*	Bacteria:*E. coli*	SCs: POPC/chol vesicles encapsulating *E. coli* (or HeLa cell) extracts	SCs:POPC/chol vesicles encapsulating *E. coli* extracts	Bacteria: *V. fischeri*	SCs:Proteinosomes made of PNIPAAm (GOx-conjugated)	Bacteria: *P. aeruginosa*	SCs:POPC/chol vesicles encapsulating *E. coli* extracts
Receiver details (specifications, preparation…)	*V. harveyi* MM32 mutant strain unable to detect AI-1 and to produce AI-2, but capable of detecting AI-2-type molecules.	*E. coli* BL21(DE3) pLysS carrying a plasmid encoding GFP under the control of a T7 promoter and a *lac* operator sequence.	Hydration of freeze-dried homogeneized liposomes, and extrusion (1 μm)	Hydration of freeze-dried liposomes	*V. harveyi* strain in which light emission is increased in response to the signal molecule produced by SCs	HRP encapsulated in the proteinosomes prepared by water droplet/oil interfacial assembly, membrane cross-linking, and phase transfer	A *P. aeruginosa* mutant stain unable to synthesize endogenous C4-HSL (*rhlI* mutant) carrying the PrhlA::lux transcriptional fusione activated by the RhlR/C4-HSL complex	Droplet transfer method (10–50 μm)
Sensor	LuxP/LuxQ sensor/transducer protein	*lac* repressor	*lac* repressor (or Tet protein)	LuxR	LuxR	Glucose oxidase	RhlR	EsaR
Response	Bioluminescence	Fluorescence (GFP)	Bioluminescence	Synthesis of a response signal: LasI-mediated AHLs synthesis from precursors (C2-HSL, SAM)	Bioluminescence (*luxA* and *luxB*)	Fluorescence (resorufin, from Amplex Red via HRP catalysis)	Bioluminescence, fluorescence (mCherry)	Synthesis and export of an antimicrobial peptide (Bac2A)
Further comments	First example of SCs sending a message to natural cells	SCs act as “translator” for natural cells	Molecular communication between two liposome-based SCs populations	Bidirectional communication between SCs and natural cells (*V. fischeri*). The article includes the study of many other systems.		Molecular communication between two different types of SCs populations (liposomes and proteinosomes)	Unidirectional communication between SCs (sender) and *P. aeruginosa* (receiver)	SCs receiver respond by producing a toxic peptide that kills the sender bacteria.

Note that the first study (column 1) is not based on gene expression. Additional examples, whereby other-than-liposome compartments are used, can be found in Schwarz-Schilling et al. (2016), Sun et al. (2016), Niederholtmeyer et al. (2018). The most representative results are shown; more details can be found in the original publications.

3OC6-HSL, N-(3-oxohexanoyl)-l-homoserine lactone; ACB, buffer containing the same components of SC interiors; AI-2, autoinducer-2; C2-CoA, acetyl-coenzyme A; C4-CoA, butyryl-coenzymeA; C4-HSL, N-butanoyl-l-homoserine lactone; C8-HSL, N-octanoyl-l-homoserine lactone; chol, cholesterol; DPhPC, 1,2-di-O-phytanoyl-sn-glycero-3-O-phosphocholine; EsaR and EsaI, signal receptor and synthase from Erwinia stewartii; GOx, glucose oxidase; HRP, horseradish peroxidase; LBS, lactobacillus selective; PNIPAAm, poly(N-isopropylacrylamide) cross-linked with glucose oxidase; POPC, 1-palmitoyl-2-oleoyl-sn-glycero-3-phsphatidylcholine; SAM, S-adenosylmethionine; Tet, ten-eleven translocation protein.

In this mini-review, the principles on which liposome-based SCs operate will be summarized, together with an explanation of the reason why they could contribute significantly to molecular communication technologies on account of their inherent possibilities in terms of design, modeling, control, programmability, and modularity. Next, recent experimental reports focused on chemical communication between SCs and natural cells (or with other SCs) will be reviewed (see also Lentini et al., 2016), while the opportunities and challenges facing this novel research arena will be discussed in the final section.

Before advancing in the discussion, two notes of warning are intended for readers unfamiliar with this research field. Firstly, the term “synthetic cell” is also used in synthetic biology to indicate *living* cells generated either by engineering biological cells (e.g., metabolic engineering, genetic optimization, or reprogramming), as well as by the transplantation of an entire synthetic genome in a living cell deprived of its own genome. Second, bottom-up synthetic biology approaches aiming at constructing cell-like systems are not restricted to liposome-based SCs. No less interesting are systems based on other types of compartments (Walde et al., 1994; Martino et al., 2012; Huang et al., 2014; Karzbrun et al., 2014; Dora Tang et al., 2015; Rideau et al., 2018), nor those based on new artificial molecules (Kurihara et al., 2011; Marguet et al., 2013; Taylor et al., 2015). Interested readers can refer to recent reviews for a broader discussion (Buddingh and van Hest, 2017; Salehi-Reyhani et al., 2017; Göpfrich et al., 2018; Schwille et al., 2018). The current review will focus only on SCs based on gene expression inside liposomes.

## Basic Principles on Liposome-Based SCs Operating Via Gene Expression

SCs based on gene expression inside liposomes find their origin in early studies on cell models aiming at achieving minimal life-like behaviors (Morowitz et al., 1988; Luisi and Varela, 1989; Schmidli et al., 1991; Oberholzer et al., 1995a,b, 1999; Szostak et al., 2001; Luisi, 2002; Pohorille and Deamer, 2002; Mansy and Szostak, 2009). Born within the origins-of-life community, this research was intended as a means of investigating the emergence of life on Earth, more precisely by demonstrating the emergence of life as a system-level phenomenon due to a particular type of organization (the autopoietic one). Hence, the *autopoietic* (self-production) (Varela et al., 1974; Luisi and Varela, 1989; Luisi, 2003) (Figure 1C) and the *chemoton* theories (chemical automaton) (Gánti, 1975) are two valuable theoretical frameworks for the construction of SCs which display features of biological organisms. Starting in the first years of 2000, SCs and similar constructs became highly relevant also in the context of synthetic biology, either as tools for generating basic knowledge, or as systems designed for applied research, i.e., biotechnology and nanomedicine.

The SCs discussed in this review are liposomes, with a size ranging typically from 0.1 to 10–100 μm: they contain DNA and a cell-free gene expression system. They are made by assembling liposomes in an aqueous phase which contains all the molecules needed to be encapsulated for accomplishing protein synthesis from a DNA template (e.g., enzymes, ribosomes, tRNAs, nucleotides, amino acids etc.). The protein synthesis machinery can derive from a cell extract or from a reconstituted system [such as the PURE system (Shimizu et al., 2001)]. Accordingly, it can be noted that SC technology is based on liposome technology (including microfluidics) and cell-free systems (including biochemical reconstitution approaches). As a result of the reactions occurring in their aqueous lumen and/or on their boundary surface, SCs can display behavior(s) typical of living cells. For example, SCs produce proteins from a corresponding gene; in turn, the synthesized protein can be an enzyme that converts substrates into products, or it can be a pore-forming protein, creating pores on the liposome membrane, or it can be a receptor that binds a signal molecule, etc. More in general, SCs can be functionalized with any chemical network of biological relevance that is functional *in vitro*.

Several reactions different from gene expression have been successfully performed inside liposomes, confirming the potentiality of SCs in terms of scope, programmability, and functionality. Some examples are: PCR and RT-PCR (Oberholzer et al., 1995a; Shohda et al., 2011; Lee et al., 2014; Tsugane and Suzuki, 2018), DNA replication (Sakatani et al., 2018; van Nies et al., 2018), and several enzymatic reactions. Moreover cytoskeletal elements have been reconstituted inside SCs (Cabré et al., 2013; Furusato et al., 2018; Litschel et al., 2018). *Ad hoc* designed gene circuits lead to SCs that can perform useful operations in a programmable way, including communication, as discussed below. SCs with the capacity of self-producing *all* their own constitutive components, and which possibly grow-and-divide as living cells do, are still missing, although interesting reports that show progress in this directions have been published (Kurihara et al., 2011).

This mini-review focuses on SCs capable of communicating with biological cells and with each other. However, other interesting research directions are under current development, including the construction of SCs with nested design (Deng et al., 2017; York-Duran et al., 2017; Hindley et al., 2018), the production of ATP inside SCs (Feng et al., 2016; Altamura et al., 2017; Lee et al., 2018), the attempts of self-producing SC parts (Schmidli et al., 1991; Kuruma et al., 2009; Scott et al., 2016; Li et al., 2017; Exterkate et al., 2018), and the shift from isolated SCs to “SC communities”, including tissue-like structures (Carrara et al., 2012; Hadorn et al., 2013; Booth et al., 2016).

## SCs That Exchange Chemical Signals: a Bottom-up Synthetic Biology Platform for Molecular Communications

SCs based on gene expression inside liposomes can be useful tools for developing molecular communication technologies (Stano et al., 2012). Current SC technology allows building simple systems capable of exchanging chemical signals, and therefore performing elementary signal processing. The idea is to design SCs capable of communicating with each other or with biological cells in a programmable manner (Figure 1D). This innovative perspective has multifold theoretical and practical consequences. From the theoretical viewpoint, SCs that can regulate their internal mechanisms in response to external perturbations (the chemical signaling) are *de facto* experimental tools for investigating minimal cognitive systems (Damiano and Stano, 2018a,b). Considering the proposed extension of the Turing imitation game to the SC realm (Cronin et al., 2006), molecular communication can contribute to the determination of life-likeness criteria as referred to SCs, as recently investigated by the Sheref Mansy group (Lentini et al., 2017). In a more practical perspective, an expansion of actual drug delivery strategies can be proposed. Inspired by the scenario depicted by Leduc and collaborators (Figure 1E) (Leduc et al., 2007), SCs could activate internal mechanisms upon perception of chemical signals, thus acting as “intelligent” drug carriers. As an example, SCs could be targeted to specific cells (e.g., tumoural cells) by exploiting antigen-antibody recognition. Once localized, their internal genetic circuit could be activated by chemical stimuli produced by the target cell itself or by other endogenous or exogenous chemical signals. These “smart” SCs could produce and release therapeutics (or drugs) *in situ*. Note that a recent study has reported SCs (injected into the tumor) that constitutively produce a toxin against breast cancer cells (Krinsky et al., 2018). The therapeutic (or diagnostic) use of SCs is, today, still a hypothetic scenario. Nevertheless, continuous improvements in SC design and construction is expected to favor a more rapid prototyping, thus accelerating the path toward applicative purposes.

### Sensors, Actuators, Controllers, and Molecular Diffusion

Like hardware robots or conventional communication devices, SCs are embodied systems composed of molecular elements that perform specific operations. Hardware components, such as sensors, controllers, and actuators (Mataric, 2007; Wang et al., 2013) have their molecular counterparts in SCs.

In the context of SCs operating by gene expression, sensors can be protein receptors or RNA aptamers that bind to a signal molecule and consequently change their conformation. This event directly or indirectly affects the “controller system,” which is based on the regulation of gene expression by protein receptors or RNA aptamers (riboswitches) at the transcriptional or translational level, respectively. These mechanisms are well-understood (Alberts et al., 2014). Depending on its design, the regulatory circuit can involve a single gene or multiple genes. As a result of this sensing-and-regulation system, the synthesis of an actuator (a protein) is promoted or inhibited. In turn, the actuator operates on some further step (e.g., producing a signal molecule, catalyzing a useful reaction, creating a pore on the SC membrane, acting as a controller/regulator of another circuit, etc.). Key examples of this general mechanism will be commented on in section A Survey of Published Reports and listed in Table 1.

To provide SCs with communication capability, water-soluble proteins (sensors, regulators, signal-producing elements, or components of the gene expression machinery) should be either encapsulated, or synthesized in the SC lumen. This has become a standard practice, somehow, at least for some prokaryotic proteins (Stano et al., 2011). It is not trivial, instead, dealing with membrane-associated and integral membrane sensors/receptors, even if reports have shown that this is a feasible goal in SCs technology (strategies as membrane protein reconstitution Yanagisawa et al., 2011; Altamura et al., 2017; Jørgensen et al., 2017 or synthesis-from-within Kuruma et al., 2009; Hamada et al., 2014; Soga et al., 2014 have been employed). Genetic circuits of distinctive complexity have already been proven to be functional, also inside liposomes (Noireaux et al., 2003; Shin and Noireaux, 2012; Siegal-Gaskins et al., 2014).

In addition to molecular elements, in order to establish an intercellular communication channel, diffusion of the signal molecule in the outer aqueous environment should be taken into account. The signal molecule cannot be directed toward the communication partner, but it spreads in all direction, guided by the concentration gradient. Although the average behavior of many signal molecules can be foreseen, individual molecules follow an erratic path. In addition to free diffusion, for closely packed SCs, communication through gap junctions (reconstituted in liposomes) has been proposed (Ramundo-Orlando et al., 2005; Moritani et al., 2010).

### A Survey of Published Reports

The pioneer experimental report on a simple cell-like system sending a signal molecule to biological cells was published by the Ben Davis group (Gardner et al., 2009). The authors encapsulated the precursors of the formose reaction inside liposomes, and observed that one class of products of the intra-vesicular reaction escaped the liposomes through a channel formed by α-haemolysin and spontaneously reacted with the borate ions present in the external medium to generate furanosyl-boronates structurally similar to the quorum sensing (QS) signal molecule AI-2, that naturally triggers bioluminescence in *Vibrio harveyi*. Remarkably, the “synthetic” signal released by the liposome was able to induce natural behavior (i.e., light emission) in this bacterium.

Despite its great interest as proof of the concept study, the SCs used by Ben Devis and co-workers were not based on gene expression, therefore they lacked those aspects of programmability and control that are peculiar to synthetic biology. Being a novel research area, literature on the liposome-based SCs which operate by gene expression to interface with natural cells (or with other SCs) is, to the best of our knowledge, limited to the six studies that are summarized in Table 1 together with the already cited study by Gardner et al. (2009). Additional cases involving non-liposome compartments are also available (Gupta et al., 2013; Schwarz-Schilling et al., 2016; Sun et al., 2016; Niederholtmeyer et al., 2018), but these will not be discussed in this mini-review.

In 2014, Sheref Mansy and collaborators designed SCs acting as “translators” for the bacterium *Escherichia coli*, using theophylline as trigger and isopropyl β-d-1-thiogalactopyranoside (IPTG) as signal molecule (Lentini et al., 2014). These SCs are liposomes containing IPTG, the PURE system as the transcription-translation (TX-TL) machinery, and a DNA template coding for a riboswitch that, after binding to the free-diffusible molecule theophylline, activated the expression of the pore forming protein α-haemolysin. The authors demonstrated that only in the presence of theophylline, did IPTG escape the liposomes through α-haemolysin, and activate the expression of the green fluorescent protein (GFP) gene in receiver *E. coli* cells. In this way, SCs acted as chemical translators allowing *E. coli* to sense theophylline (the latter molecule cannot be normally sensed by *E. coli*).

Adamala et al. (2017) built SCs containing engineered genetic circuits and regulatory cascades. These SCs can be controlled/triggered by external signals, and can be fused together in order to bring together products of incompatible reactions. In particular, the group lead by Edward Boyden showed that by using cell lysates with transcriptional-translational activity, DNA vectors encoding genes for IPTG (or doxycycline) detection and permeable chemical inducers, as arabinose or theophylline, the arabinose (or theophylline) activates the α-haemolysin production in the first SC population, so that pre-encapsulated impermeable IPTG (or doxycycline) could be released, and thus activate a response in a second SC population.

The group of Sheref Mansy recently reported two-way chemical communication between SCs and bacteria (Lentini et al., 2017). They exploited cell extracts to generate SCs able to synthesize molecules perceived by *Vibrio fischeri, V. harveyi, E. coli*, and *Pseudomonas aeruginosa*. In particular, the expression of LuxI-like synthases inside liposomes, in the presence of acetyl coenzyme A and *S*-adenosylmethionine (SAM), resulted in the production of molecules able to activate *E. coli* and *V. fischeri*-based biosensor strains for acyl-homoserine lactone (AHLs) detection. Cell extracts operated both for TX-TL reactions and for the synthesis of some AHL precursors. Moreover, it was shown that SCs containing *ad hoc* designed genetic circuits could express QS signal molecule receptors able to trigger the expression of reporter and QS signal synthase genes (e.g., *gfp* and *luxI*), upon perception of QS signal molecules produced by bacteria. The extent to which SCs could “imitate” natural cells in term of their response to the investigated QS signal molecule was estimated by a sort of cellular Turing test (Cronin et al., 2006).

The signaling between liposome-based SCs and proteinosomes (cell-like particles made of proteins) mediated by glucose, has been recently reported by a joint work of the groups of Sheref Mansy and Stephen Mann (Tang et al., 2018). In this study, the unidirectional signaling pathway was based on: (i) liposome transmitters, containing the PURE system, a DNA plasmid carrying a chemically inducible repression switch (EsaR), a gene coding for α-haemolysin, and glucose; (ii) proteinosome receivers, consisting of a cross-linked enzymatically active glucose oxidase (Gox)-poly(*N*-isopropylacrylamide) (PNIPAAm) membrane and encapsulated horseradish peroxidase (HRP). The addition of the permeable AHL molecule *N*-(3-oxohexanoyl)-l-homoserine lactone (3OC6-HSL) triggered intravesicular α-hemolysin expression and consequent membrane pore formation in liposome-based SCs, which allowed the release of glucose contained in the aqueous lumen. Glucose oxidation on the proteinosome membrane produced hydrogen peroxide, which in turn converted a molecule into a fluorescent output by reacting with the HRP encapsulated in proteinosome. This study provides an example of molecular communication between two different types of artificial cell-like systems.

A recent report comes from our laboratory, and it deals with unidirectional SC–*P. aeruginosa* communication, based on the QS AHL signal molecule C4-HSL (Rampioni et al., 2018). In particular, SCs were prepared by encapsulating the PURE system inside GVs prepared by the droplet transfer method (Pautot et al., 2003; Fujii et al., 2014), together with butyryl coenzyme A and SAM as precursors, and a plasmid encoding for RhlI, the synthase for C4-HSL production. SCs produced C4-HSL (a natural QS signal molecule), which was perceived by *P. aeruginosa* both in liquid medium and in gel. In particular, *P. aeruginosa* modified its gene expression pattern in response to the C4-HSL-produced by SCs, demonstrating that reprogramming of gene expression in the bacterial cell is similar when interacting with other bacteria or with SCs. The entire TX-TL mechanism was assessed by *rhlI* mRNA and RhlI protein quantification, as well as by chemical identification of the C4-HSL signal produced by the SCs. The experimental results interestingly match with previously published numerical modeling (Rampioni et al., 2014), confirming the predictive power of *in silico* simulations in SCs research.

Finally, the Tan group reported an interesting study where SCs and bacteria engaged unidirectional communication in various ways (SCs to SCs, bacteria to SCs, and SCs to bacteria) (Ding et al., 2018). In this case, the QS signal molecule was produced *via* the EsaI synthase, and was perceived by the cognate EsaR receptor. Gene expression in SCs was triggered when binding of the QS signal molecule to EsaR led to derepression of an EsaR-controlled promoter region. Quite interestingly, the authors designed SCs that produce an antimicrobial peptide (Bac2A) in response to QS signal molecules sent by bacteria—a proof of principle of the use of signal processing and actuation dynamics for the generation of SCs interfacing with natural cells. Moreover, SCs embedded in biofilms were also reported.

## Directions and Challenges for Future Work

The works compared in Table 1 represent proof-of-concept pioneer works that will likely stimulate further research to expand SC capabilities related to molecular communications. In this context, several challenges and open questions can be envisaged. Some refers to mechanistic, biochemical and biological aspects, others to the capability of engineering molecular communications.

With respect to the mechanisms of molecular communication, “sender” and “receiver” SCs mainly relied on transmembrane diffusion of signal molecules. This simple approach has been effective because some QS signal molecules, such as short-tail AHLs, can cross the lipid bilayer (Pearson et al., 1999). The generation of α-haemolysin pores is a drastic (yet effective) solution that has been used to bypass the low permeability of SC membranes when non free-diffusible signal molecules have been used (e.g., IPTG or glucose), but this causes the release of all the low-MW compounds contained inside SCs (the cut-off molecular weight value for the α-haemolysin pore is 3 kDa; Song et al., 1996). An alternative could be the use of DNA nanopores, whose properties are tunable by design (Krishnan et al., 2016). The future employment of more sophisticated import/export mechanisms based on membrane proteins will allow expanding the chemical repertoire of signal molecules secreted or perceived by SCs (e.g., peptides), thus increasing the communication capability and specificity. In this respect, ongoing progress on the functionalization of SC envelopes with integral membrane proteins is promising (see section Basic Principles on Liposome-Based SCs Operating via Gene Expression).

Looking at the biological partners of SCs for molecular communications, early studies focused on bacteria, since they are prone to genetic engineering and their intercellular communication systems have been thoroughly studied at the molecular level, especially in the case of QS systems. From a practical viewpoint, SC/bacteria communication is a technological platform for the long-term goal of interfering with bacterial populations and for therapeutic strategies that could be devised against infections. Indeed, the ability of SCs to drive gene expression in response to external cues envisages the generation of injectable SCs endowed with the ability to produce or release an antimicrobial compound only in response to a signal molecule produced by a bacterial pathogen. The study reported in Table 1 by the Tan group (Ding et al., 2018) has provided a proof-of-principle that SCs can be generated which are able to kill bacteria by a mechanism triggered by the bacteria themselves.

Proving that SCs can communicate with eukaryotic cells is one of the next milestones, especially when nanomedicine applications are devised. This complex task could require the generation of SCs with internal operations that rely on eukaryotic signal synthesis or more complex signal reception machineries. The relevance of these approaches is that SCs could be employed as intelligent drug-delivery systems that perform a therapeutic action by extracting information from their microenvironment. As mentioned, the generation of SCs constitutively producing a tumor-killing protein (the *Pseudomonas* exotoxin A) has been recently described (Krinsky et al., 2018). Another task would involve enzyme replacement therapy (Itel et al., 2017). For example, SCs that consume excess phenylalanine could play a therapeutic role in phenylketonuria (Leduc et al., 2007). Notably, Thomas M. S. Chang proposed in a pre-liposome age the therapeutic use of enzyme-containing semi-permeable collodion capsules circulating in the bloodstream (Chang, 1964, 1972). The generation of SC interfacing *via* molecular communication with neural cells can also be imagined. The resulting hybrid bio/synthetic cell networks could also be exploited for innovative investigations of neural functions (Pinato et al., 2011).

Considering the engineering plan of networking SCs (or SCs and biological cells), the rigorous design of molecular communication channels requires a proper modeling of the physical and information levels. At the physical level stochastic diffusion plays a central role. This peculiar aspect is the ultimate limit of molecular communication (when compared to traditional electro-magnetic systems) because it is essentially a random process. Intercellular molecular communications rely on diffusion of chemical signals under a concentration gradient. They are, therefore, slow stochastic processes; their success depends on a number of factors, like the sender/receiver ratio, their spatial arrangement, the viscosity of the medium, and the temperature. Numerical models can be useful to understand the limiting factors and the constraints operating at this (inescapable) physical level (Nakano et al., 2011, 2013). The stochastic dimension of molecular communications affects its reliability. Facing with it represents an engineering challenge. The second aspect refers to the amount of information transmitted in the molecular communication “channel,” and this is a theoretical issue. To apply classical information and communication theory to such a novel scenario, “information” should be defined with respect to the type of signal molecules, number of sent/received molecules, and time-dependent concentration profile (switch-like, pulse-like, etc.). Control theory for bottom-up synthetic biology should be delineated (Del Vecchio et al., 2016). Its peculiarity stems from molecular discreteness, random timing of sending/receiving, nature of “noise,” etc.

In conclusion, SCs could significantly contribute to the origin of a very novel research field based on communication with biological cells. Thanks to their modular constructive principle, their biocompatibility and programmability, SCs of the type discussed in this review have the unique ability to act as passive carriers of hydrophilic and hydrophobic drugs, and to actively drive gene expression in response to chemical stimuli from other cells and from the environment.

At present, main challenges in this field rely on our capacity of (i) designing and build multi-functional SCs based on a proper genetic circuit and auxiliary molecular parts/devices, (ii) building homogeneous populations of SCs that are stable in biological fluids, (iii) and being able to control SC behavior even in a complex and fluctuating environment, such as a human host. All these challenges will probably be solved in the near future thanks to constant improvements in SC technology (in a broad sense, i.e., not necessarily restricted to liposomes). Along this path, there will be room for developing various systems in which *in vitro* usage will generate opportunities for understanding principles of biological systems and constructing short-term devices (e.g., biosensors).

## Author Contributions

PS conceived the research, all authors wrote the paper.

### Conflict of Interest Statement

The authors declare that the research was conducted in the absence of any commercial or financial relationships that could be construed as a potential conflict of interest.
